# RelB and Neuroinflammation

**DOI:** 10.3390/cells10071609

**Published:** 2021-06-27

**Authors:** Karli Mockenhaupt, Alexandra Gonsiewski, Tomasz Kordula

**Affiliations:** Department of Biochemistry and Molecular Biology, School of Medicine and the Massey Cancer Center, Virginia Commonwealth University, Richmond, VI 23298, USA; Karli.Mockenhaupt@vcuhealth.org (K.M.); krebsa@vcu.edu (A.G.)

**Keywords:** neuroinflammation, NF-κB, RelB, astrocytes, microglia, oligodendrocytes, glioblastoma multiforme

## Abstract

Neuroinflammation within the central nervous system involves multiple cell types that coordinate their responses by secreting and responding to a plethora of inflammatory mediators. These factors activate multiple signaling cascades to orchestrate initial inflammatory response and subsequent resolution. Activation of NF-κB pathways in several cell types is critical during neuroinflammation. In contrast to the well-studied role of p65 NF-κB during neuroinflammation, the mechanisms of RelB activation in specific cell types and its roles during neuroinflammatory response are less understood. In this review, we summarize the mechanisms of RelB activation in specific cell types of the CNS and the specialized effects this transcription factor exerts during neuroinflammation.

## 1. Introduction

Neuroinflammation is the homeostatic defense mechanism that is activated in the central nervous system (CNS) and aims at preventing exacerbated damage when faced with insults such as injury, infection, autoimmune response, or metabolic stress [1,2,3]. The innate and adaptive immune systems are activated in response to these insults [4,5,6]. The innate immune system is quicker to react but is nonspecific and is composed of innate immune cells, including the resident microglia of the brain and bone-marrow-derived monocytes/macrophages [6]. The adaptive immune system, which depends on activation of B cells and T cells, is much more specific but requires time to ramp up [4]. Nevertheless, the CNS is separated from the rest of the body by a blood–brain barrier (BBB) that includes both endothelial cells and astrocytes, limiting the entry to the CNS [7]. Therefore, T cell entry is tightly restricted, especially at the onset of neuroinflammation [7]. In addition to the immune cells, other cells, such as astrocytes and oligodendrocytes, modulate the immune response within the CNS [8,9,10,11]. Unresolved chronic neuroinflammation can lead to neurodegeneration, which manifests by a gradual obliteration of neuronal cells. Neurodegeneration embodies the pathologies of several debilitating diseases, including multiple sclerosis (MS), Alzheimer’s disease, Parkinson’s disease, Huntington’s disease, and amyotrophic lateral sclerosis, among others [12,13,14]. Neurodegeneration compiles both molecular and cellular events that include an accumulation of protein aggregates, modified mitochondria functions, oxidative responses, and cell death [1,15,16,17,18,19]. Although multiple transcription factors regulate neuroinflammatory responses (reviewed in [20,21]), this review is concentrated on RelB, which is a member of the nuclear factor kappa B (NF-κB) family of transcription factors.

## 2. The NF-κB Signaling Pathways

**Figure 1:**
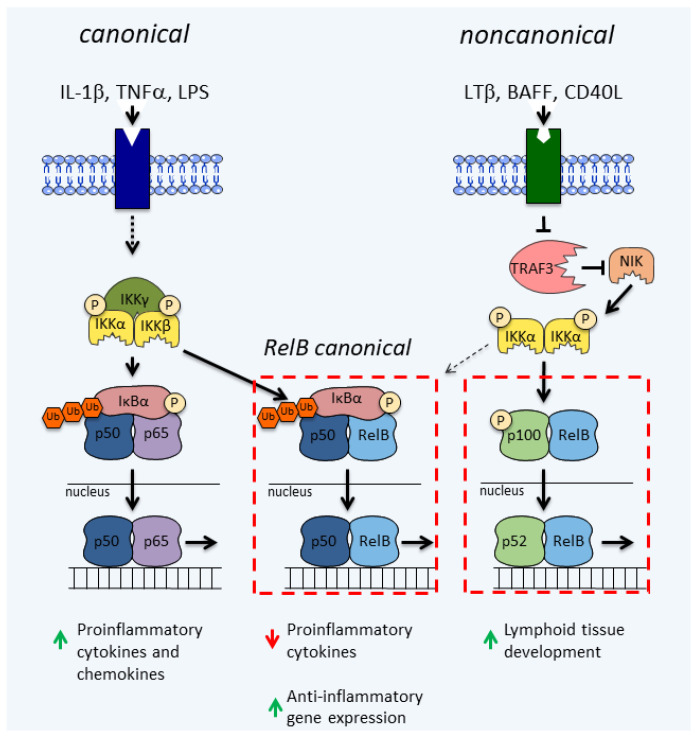
Activation of RelB by the nuclear factor kappa B (NF-κB) signaling pathways. The canonical NF-κB pathway is activated by interleukin 1β (IL-1 β), tumor necrosis factor α (TNFα), and liposaccharide (LPS). The noncanonical NF-κB pathway is activated by lymphotoxin β (LTβ), B cell activating factor of the TNF family (BAFF), and CD40 ligand (CD40L). The canonical activation of RelB/p50 occurs in cells expressing high levels of RelB (basally or after induction). IκB, inhibitor of NF-κB; IKK, IκB kinase; NIK, NF-κB-inducing kinase; P, phosphate; and TRAF3, TNF receptor-associated factor 3.

The NF-κB family of transcription factors includes p65 (RelA), c-Rel, p105/p50 (NF-κB1), p100/p52 (NF-κB2), and RelB, which can be activated by different ligands via distinctive signaling pathways that have been extensively studied over the last several decades (reviewed in [22,23,24]). The canonical NF-κB pathway is triggered by an array of inflammatory stimuli, including proinflammatory cytokines (i.e., tumor necrosis factor α (TNFα) and interleukin-1β (IL-1β)), molecules recognized by the pattern-recognition receptors (i.e., Toll-like receptor (TLRs) ligands), and antigens, among others [23,24,25,26]) (Figure 1). The engagement of the canonical pathway rapidly activates the inhibitor of κB kinase (IKK) complex, which is made up of three subunits: IKKα (also known as IKK1), IKKβ (IKK2), and IKKγ (also known as NF-κB essential modulator (NEMO)) [27]. Activated IKKβ subsequently phosphorylates the inhibitor of NF-κB (IκB) proteins, including IκBα, which are subsequently ubiquitinated and degraded by the proteasome [28]. This releases the p65/p50 and c-Rel/p50 complexes that enter the nucleus and induce transcription of hundreds of target genes, including those encoding major proinflammatory cytokines and chemokines as well as IκBα and RelB [22,29]. While inflammatory cytokines and chemokines recruit immune cells at the local sites of inflammation, IκBα and RelB provide a negative feedback loop needed to limit the initial activation.

The noncanonical pathway (reviewed by [30]) is induced by a much more limited set of ligands that bind to their receptors, which include B cell activation factor receptor (BAFFR), lymphotoxin β receptor (LTβR), cluster of differentiation 40 (CD40), receptor activator of NF-κB (RANK), and fibroblast growth factor-inducible 14 (Fn14) [26,31,32,33,34]. In cells expressing NF-κB-inducing kinase (NIK), the TNF receptor-associated factor (TRAF) 3 forms a complex with TRAF2, the cellular inhibitor of apoptosis (cIAP) 1, cIAP2, and NIK, and this leads to constitutive ubiquitination and degradation of NIK [35]. When the ligands of the noncanonical pathway bind, they induce the recruitment of TRAF3 to their receptors and its subsequent degradation [36]. Simultaneously, the released cIAP1/cIAP2/TRAF2 complex no longer can interact with NIK leading to its accumulation. Accrued NIK phosphorylates and activates IKKα [37,38], which in turn phosphorylates p100 (sometimes referred to as IκBδ) [28]. This results in the processing of p100 into p52 [38], formation of the RelB/p52 heterodimers [39], translocation of these complexes to the nuclei, and induction of specific gene transcription, including those regulating lymphoid tissue development [40]. In contrast to rapid activation of NF-κB-dependent genes by the canonical pathway, activation of the noncanonical pathway is much slower and more persistent. Interestingly, in cells expressing high levels of RelB, LTβ also induces the formation of RelB/p50 dimers that contribute to the development of Peyer’s patches [26], which are clusters of lymphoid tissue located in the small intestine and regulate the intestinal flora.

**Figure 2:**
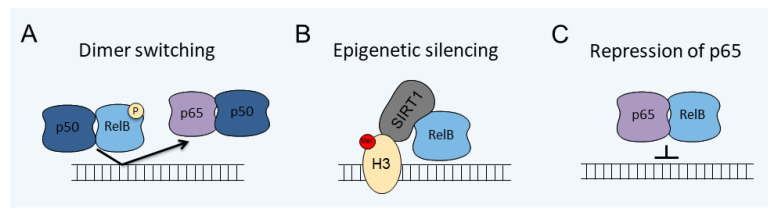
The mechanisms of RelB inhibition of p65/p50-mediated gene expression. (**A**) Dimer switching involves replacement of the p65/p50 complexes by the RelB/p50 complexes. (**B**) Epigenetic silencing involves recruitment of SIRT1, histone H3 deacetylation, and methylation. (**C**) Direct binding of p65 by RelB inhibits DNA binding. Histone H3, (H3); methyl, (Me); phosphate, (P); and Sirtuin 1, (SIRT1).

The least studied is the activation of RelB by the canonical pathway, which is limited to some cell types, such as dendritic cells, and combines factors from both the canonical and noncanonical pathways (RelB canonical pathway) (Figure 1). Similar to the canonical pathway, the RelB canonical pathway is stimulated by IL-1β, TNFα, and LPS [41,42] and has the same upstream factors [43]. In the cytoplasm, the RelB/p50 heterodimers can form complexes with IκB proteins [43]. This requires high levels of RelB expression due to the higher affinity of RelB to p52 than p50 [44,45], which limits this activation, in normal physiological conditions, to only some cell types, such as dendritic cells [46]. Although nonlymphoid cells, including astrocytes, express RelB at low levels, in response to inflammatory stimuli, such as IL-1β, levels of RelB are dramatically upregulated, which induces the formation of the RelB/p50/IκBα complexes [42,47]. The RelB/p50 complexes are activated by the canonical pathway, translocate to the nucleus, and initiate the expression of responsive genes, including anti-inflammatory genes, such as YKL-40 [42] and IκBα [48]. The RelB/p50 complexes also limit expression of the proinflammatory cytokines (Figure 2A) [47]. In myeloid cells, the RelB/p50 complexes were shown to also limit cytokine expression, however, by a different mechanism involving epigenetic silencing (Figure 2B) [40,49]. It has also been proposed that RelB inhibits inflammatory responses by directly binding p65, forming a transcriptionally inactive complex, and thus limiting activation of p65/p50-dependent genes (Figure 2C) [50].

## 3. RelB

The gene encoding RelB is located on the human chromosome 19q13.32 and has recently been shown to encode 12 exons making up a protein with 579 amino acids [51]. The promoter region of RelB is unique and does not have a TATA-binding region but contains two κB binding sites [52]. While high basal expression of RelB is limited to dendritic cells [46], proinflammatory stimuli rapidly induce RelB expression in immune cells, including T cells, B cells, and monocytes as well as other cell types, such as astrocytes [42,47,53,54].

RelB is composed of three distinctive domains. The Rel homology domain (RHD) is shared by all five NF-κB members and is involved in dimerization, nuclear translocation, and DNA binding [44,55]. However, unlike all the other NF-κB members [56,57,58,59,60,61], RelB is unable to form homodimers [55]. Importantly, RelB forms heterodimers with p50/p105 [62] and p52/p100 [45] and also p65 [63]. The transcriptional activation domain (TAD) is only shared between RelB, p65, and c-Rel and is essential but not sufficient for transcriptional activation of the NF-κB target genes [64,65]. The leucine zipper (LZ) domain that resides on the N-terminus is unique to RelB and is thought to allow RelB to bind to more diverse consensus sequences [66,67], but the exact function of this domain remains to be defined.

Like other NF-κB members, regulation of RelB is fine-tuned by post-translational modifications, including polyubiquitination, SUMOylation, and phosphorylation (Reviewed in [68]). For example, RelB is destined for degradation by Thr84 and Ser552 phosphorylation [69]. While the kinase responsible for Thr84 remains unknown, glycogen synthase kinase-3β (GSK-3β) mediates Ser552 phosphorylation [70]. Additionally, the IKK complex phosphorylates RelB at Ser472 in response to TNF-α or IL-1β [47,71]. The RelB/p50 complexes containing Ser472 phosphorylated RelB induce the expression of genes associated with migration of fibroblasts, such as matrix metallopeptidase 3 (MMP3) [71]. This phosphorylation was also shown to be critical for limiting cytokine gene expression in astrocytes [47]. It has been proposed that the Ser472 phosphorylation decreases association with IκBα; however, this is still debated [47,71]. A fourth RelB phosphorylation site was identified at Ser368, which is critical for the dimerization of RelB with p100 and blocks the cleavage of p100 to p52 [63]. Additional putative RelB phosphorylation sites have been identified by mass spectrometry, but their functions in vivo remain unknown [68].

Large amounts of data implicate RelB in immune functions [24,40]. RelB is most well-known for its critical function in the noncanonical pathway, controlling lymphoid organ development [31]. Global RelB knockout mice have a range of immune deficiencies, including impaired development of Peyer’s patches [31], germinal centers [72], and the medullary epithelium [72] (reviewed by [73]). Further, RelB plays a critical role in the differentiation of dendritic cells [74], secondary lymphoid tissue organization, and osteoclasts [75]. These RelB-dependent functions are also dependent on p52 and localized to the sites of increased basal RelB expression [31,72]. However, RelB knockout mice have much more significant deleterious effects than the NIK knockout mice, suggesting additional roles of RelB that are independent of the noncanonical pathway [76]. The RelB knockout mice also have a shorter lifespan due to noninfectious multiorgan inflammatory syndrome that is T-cell dependent but independent of B cells [77].

Interestingly, it has been proposed that RelB stifles expression of proinflammatory genes in myeloid cells during the late phase of septic shock, thus providing an important negative feedback loop [78]. In mouse models of endotoxin tolerance, RelB works with Sirtuin 1 (SIRT1) to coordinate an epigenetic switch, silencing proinflammatory gene expression, including genes encoding TNFα and IL-1β [49,78,79] (Figure 2).

### 3.1. RelB in the CNS

RelB has been found to potentially play a role in a variety of CNS diseases. First, intracerebral hemorrhage induces expression of all members of the NF-κB family, including RelB [80]. Second, chronic hyperglycemia-induced oxidative stress also activates NF-κB signaling with induction of p65, RelB, and p50 in the hypothalamus, basolateral amygdala, and cerebral cortex [81]. Third, p65, RelB, and p52 undergo nuclear translocation in a mouse model of Parkinson’s disease that destroys dopaminergic neurons in the substantia nigra [82]. Although RelB forms dimers with estrogen receptor beta (ER-β), the effect of these dimers on neuroinflammation remains unknown [82]. Fourth, APOE ε4 allele is a known genetic risk factor for the late-onset Alzheimer’s disease [83,84]. Two independent studies identified RelB gene variants that associate with APOE ε4 [85,86]. Rare RelB variants associated with amyloid burden in the frontal and parietal lobes and the hippocampus [85,86]. While evidence accumulates on the expression, interacting partners, kinetics, and functions of RelB in the CNS, additional studies are needed to determine whether RelB could be targeted for future therapy.

### 3.2. RelB in Microglia

Microglia are the long-lived resident immune cells of the brain [87]. However, unlike short-lived macrophages which originate in the bone marrow, microglia stem from myeloid precursors in the yolk sac [88,89]. Their motile processes provide constant surveillance of the local microenvironment. Microglia express a wide array of pattern recognition receptors that enable rapid detection of pathogens and cell debris [90,91,92]. When responding to injury or disease, microglia acquire phenotypes that range from pro- to anti-inflammatory [93]. Depending on the phenotype, microglia release a plethora of cytokines, chemokines, growth factors, and other pro- and anti-inflammatory molecules [91,94]. Further, microglia have critical phagocytic properties that are required for removing debris, including apoptotic cells, in both the healthy and diseased brain [95].

Similar to macrophages [96], microglia display adaptive responses to subsequent infections and inflammatory encounters [97]. After a stimulus, such as LPS, the immune cells may become preprogrammed for a subsequent stimulus [5,98]. When the initial dose of LPS is low, immune cell training (also known as priming) occurs, resulting in increased response to subsequent stimulation [98]. Directly contrasting this, immune cell tolerance follows pre-exposure to higher doses of LPS, which limits microglia response upon re-exposure [98].

It has been elegantly shown that tolerance in microglia is mediated by RelB [99]. Interestingly, tolerized microglia display a blunted immune response with reduced cytokine production, but they increase their phagocytic activity and secretion of inducible nitric oxide synthase (iNOS), retaining the properties that resolve inflammation [99]. Although the detailed mechanism remains unknown, tolerance in microglia involves epigenetic alterations to the chromatin of targeted loci (Table 1). An increase in the dimethylation of histone H3 on lysine 9 (H3K9me2), which is a silencing modification, has been observed [99]. Interestingly, RelB-dependent tolerance also occurs in monocytes. It has been proposed that RelB binds with histone H3K9 methyltransferase G9a, which is critical for silencing [49]. Microglial tolerance is long lived, lasting at least six months [98], which may be due to microglia’s long lifespan [100,101] and important in the prevention of excess CNS damage. Further, microglia tolerance is not limited to LPS re-exposure as tolerance also protected against Alzheimer’s pathology and ischemia, although the role of RelB has not been directly studied in those cases [98].

RelB was also proposed to suppress proinflammatory pathways in human immunodeficiency virus-1 (HIV-1)-associated neurocognitive disorder (HAND) [102]. HAND is thought to be induced by inflammation and oxidative stress mediated by the transactivator of transcription (Tat) protein [103]. In microglia, Tat induces the expression of RelB and TNF-α [102]. RelB counteracts inflammation through anti-inflammatory pathways and provides a negative feedback loop against p65/p50 activation [102]. Altogether, RelB works to suppress proinflammatory immune responses in microglia.

### 3.3. RelB in Astrocytes

Although astrocytes were originally thought to be only the supporting cells of the CNS, recent data clearly demonstrate that these cells are critical regulators of many processes in the CNS [109,110]. Astrocytes support neurons, maintain ion balance, support the blood–brain barrier, regulate water transport, reinforce and prune synapses, guide migrating neurons, regulate concentrations of neurotransmitters, and aide with immune functions [109,110,111,112,113]. However, when astrocytes become reactive, they undergo morphological and functional changes, reprogram gene expression profiles, and secrete and respond to a plethora of inflammatory mediators [112,114]. These responses may be beneficial or harmful and are heterogeneous, depending on the phenotypic polarization of astrocytes, their diversity, and the type and magnitude of the stimulus [115].

In cultured human astrocytes, RelB is expressed basally at low levels [42,47]. After stimulation with IL-1β, the p65/p50 complexes translocate to the nuclei and induce transcription of target genes, including proinflammatory cytokines and chemokines, as well as RelB [47]. Once the RelB protein is synthesized, it is phosphorylated on Ser472 by IKK-dependent mechanisms [47]. Similarly to tolerance in microglia, tolerance has also been observed in astrocytes [47,116]. The proposed mechanism of tolerance in astrocytes involves RelB; however, it is different than the one described for microglia and monocytes [78,99]. Tolerance in astrocytes occurs after p65/p50 dimers are stripped from the DNA by resynthesized IκBα that enters the nuclei. The previously proposed dimer switching [117] occurs with p65/p50 dimers being replaced by the RelB/p50 complexes [47]. It is speculated that RelB Ser472 phosphorylation prevents the stripping of RelB/p50 complexes from the DNA by resynthesized IκBα, allowing perdurance of RelB/p50 on the cytokine promoters [47]. Indeed, this phosphorylation persists in astrocytes for several days. However, RelB is also known to stabilize p100 [63,118], which could limit p65/p50 activation. Similarly to microglia, RelB was shown to bind SIRT1 in astrocytes [47]. This suggests that SIRT1 may be responsible for deacetylation of p65, allowing its removal from the DNA as observed in other cell types [119]. In contrast to microglia [99], epigenetic changes at RelB-regulated cytokine promoters have not been observed in astrocytes. Additionally, tolerance in these cells last for days [47], which differs from long-lasting tolerance of microglia [99]. Importantly, astrocytic RelB also controls tolerance in vivo in a mouse model of systemically induced neuroinflammation [47].

Not surprisingly astrocytic RelB expression is also increased during experimental autoimmune encephalomyelitis (EAE), a mouse model of multiple sclerosis (MS) [120]. Its expression is also downregulated in human astrocytes in vitro by a drug used to treat relapsing-remitting MS, isosorbide dimethyl fumarate (IDMF) [121]. However, conditional deletion of RelB from astrocytes in mice only delays the onset of EAE but does not protect the mice from the disease [104]. This observation confirms the known role of astrocytes in the pathogenesis of EAE and MS but also indicates that multiple cell types contribute to the disease.

### 3.4. RelB in Oligodendrocytes

Oligodendrocytes are the myelinating cells of the brain and spinal cord that create an insulating sheath around axons in a concentric fashion [122,123]. The myelin sheath accelerates rapid transmission of action potentials due to its low capacitance. Oligodendrocytes also provide metabolic support by generating lactate for energy, have an immunomodulatory capacity, and provide trophic factors [123,124]. Interestingly, oligodendrocytes can be differentiated in the adult brain from oligodendrocyte precursors cells (OPCs) by exiting the cell cycle and producing myelin proteins [125]. The myelination in the adult can be controlled by neuronal activity and is critically regulated by epigenetic modifications [125,126].

In the disease pathology of both MS [127] and EAE [128], oligodendrocyte death is followed by the regeneration by OPCs, which is a main determinant of clinical prognosis (reviewed by [129]). However, microglia and astrocytes are the primary producers of the inflammatory mediators, not the oligodendrocytes (reviewed by [130]). It has been shown that p65 aids in survival of oligodendrocytes in vitro [131,132,133]. However, constitutively active IKKβ in oligodendrocytes of mice had little effect on the survival and functions of oligodendrocytes under normal conditions [134]. By contrast, during EAE, these mice had reduced demyelination, oligodendrocyte death, and axon degeneration, resulting in overall decreased disease severity [134]. This phenotype was attributed to the activation of p65 and increased p65-dependent oligodendrocyte survival [133,134]. Interestingly, in a mouse expressing a IκBαΔN super-repressor (lacking N terminus and thus resistant to proteasomal degradation) in oligodendrocytes, there was no effect on demyelination or remyelination in a cuprizone model of MS [133]. However, additional interferon γ treatment induced more severe disease in these mice [133]. Similarly to these findings, decreased EAE severity has been reported in oligodendrocyte-specific RelB conditional knockout mice [104]. This was attributed to prolonged activation of p65 and increased oligodendrocyte survival [104]. Although the exact mechanism remains elusive, p65 is known to control expression of several antiapoptotic genes including cIAPs, cellular FLICE-inhibitory protein (cFLIP), Bcl-2, Bcl-xL, TRAF1, and TRAF2 [135,136]. Ultimately and in contrast to the role of RelB that limits expression of inflammatory mediators in microglia and astrocytes, RelB limits p65-dependent oligodendrocyte survival [104,134].

### 3.5. RelB in Neurons

Neurons, electrically excitable cells of the CNS, are represented by a large number of highly specialized subtypes [137]. These cells create specific neural circuits through the expansion of axons, prevention of collision of axons and dendrites, specification of axon-dendrite partners, and creation and refinement of synapses [138,139,140]. A variety of molecular mechanisms define the programs that generate neuronal development, including neuronal cell lineage, timed cell division, contact inhibition, secreted factors, and lateral inhibition [141].

Although NF-κB signaling is important for neuronal survival, the role of RelB has not been decisively established [142,143]. A novel reporter mouse identified that RelB is expressed in the nervous system in the postnatal and adult brain [144]. While the specific cell types expressing RelB were not identified, RelB was absent from the myelin tracts and was speculated to be expressed by neurons [144]. This finding supports previous data indicating enriched RelB expression in the synaptosome fraction [145]. Additionally, constitutive NF-κB activity is required for the survival of neurons [146]. It has been proposed that the neuroprotective effects of glial cell line-derived neurotrophic factor (GDNF) on dopaminergic neurons is NF-κB-dependent [147]. These cells express NIK and GDNF induces IKKα-mediated phosphorylation of p100 and its subsequent processing to p52 [147]. Surprisingly, however, it was proposed that the p65/p52 heterodimers translocate to the nucleus and block apoptosis, and this process is independent of RelB [147]. Of note, LTβ induces gliogenesis of neural progenitor cells/neural stem cells primarily through activation of the canonical NF-κB pathway and also induces RelB, but the implications of RelB activation remain unknown [148]. NF-κB signaling is also critical for maintaining neural cell integrity in the brain and retina during oxidative stress, ischemic stroke, and neurodegeneration [149]. Neuroprotectin D1 (NPD1) aids with cell survival by inducing the expression and activation of c-Rel, which stimulates RelB expression independently of the canonical pathway [149]. However, the direct implications of RelB on maintaining neural integrity remain elusive.

### 3.6. RelB in Other Cells of the CNS

The role of RelB in other cell types of the CNS remains largely unexplored. It is accepted that NF-κB activation in endothelial cells observed during neuroinflammation contributes to BBB dysfunction [150]. In addition to endothelial cells, pericytes are essential in blood vessel formation, BBB maintenance, and regulation of leukocyte infiltration [151,152]. In a model of diabetic retinopathy, IL-1β secretion from microglia and endothelial cells results in the activation of NF-κB in pericytes, inducing apoptosis and reducing the number of tight junctions [153]. Further, RelB is critical for lymphatic vessel maturation and function that is not dependent on p52 [154]. However, the role of RelB in brain endothelial cells and pericytes remains elusive.

Dendritic cells are antigen-presenting cells that survey the environment, including the CNS. The expression of RelB in dendritic cells is essential for the quantitative regulation of Tregs [155]. Deletion of RelB from dendritic cells induces an influx of Tregs and protects mice from EAE [155]. Interestingly, deletion of gene encoding p100/p50 only partially recapitulates this phenotype, suggesting that the noncanonical pathway is not fully responsible for Treg accumulation [155]. 

## 4. The Immunosuppressive Role of RelB in GBM

Glioblastoma multiforme (GBM) is the most common type of primary brain cancer in adults, and a stage IV glioma as classified by the world health organization [156]. GBM has a poor survival rate with a median survival of only fifteen months even after resection, radiation, and chemotherapy [157,158,159]. GBM tumors are extremely invasive and extensively angiogenic and necrotic [160,161]. Additional characteristics of GBM include inter- and intra-tumor heterogeneity [162,163,164], regions of blood–brain barrier (BBB) disorganization (reviewed by [165]), malignant neovascularization [166], and immunosuppressive inflammation that inhibits antitumor responses and promotes GBM growth and progression [166,167]. While all these characteristics make GBM difficult to treat, the high levels of immunosuppressive inflammation have proven particularly challenging to combat.

Ultimately, three main pathways are often altered in GBM, including p53 signaling, retinoblastoma (RB)-mediated cell-cycle control, and receptor tyrosine kinase (RTK) signaling [168,169]. While originally four different molecular GBM subtypes were identified based on unsupervised gene clustering [170,171], recently three major subtypes, proneural, classical, and mesenchymal have been recognized [163,171,172]. The proneural subtype frequently has PDGFRA amplifications, as well as mutation in IDH1 and TP53 [171]. The classical subtype often has EGFR abnormalities, and the mesenchymal subtype displays alterations to NF1 and also increased RelB expression [171,173]. Extensive studies indicate the oligodendrocyte precursor cell as a cell of GBM origin [174,175,176,177]. The immune landscape also differs across GBM subtypes with the largest infiltration of microglia, macrophages, and lymphocytes in the mesenchymal subtype [178], while the classical subtype contains a significant number of astrocytes [163,179]. This complicated network of cells in the GBM microenvironment is a key factor contributing to the immunosuppressive inflammation [109]. Glioma-associated microglia/macrophages (GAMs) make up to 30% of the GBM tumor mass, aide in tumor proliferation and invasion, and correlate with poor prognosis [158,178,180,181]. GAMs exhibit a complex immunosuppressive phenotype [182] and express both anti- and pro-tumorigenic factors normally expressed by M1 and M2 macrophages and microglia. GAMs also express an array of other factors such as VEGFA, TGFβ, and metalloproteases [183,184,185,186,187]. By contrast, lymphocytes compose less than 5% of the tumor microenvironment [178]; however, increased numbers of Treg cells infiltrating the tumor have been found in GBM patients [188,189].

In addition to immune cells, astrocytes that contact GBM cells encompass the tumor, become reactive, and alter their gene expression, including increased GFAP expression, which is historically used to visualize them (reviewed by [190]). These reactive astrocytes promote cell proliferation and infiltrative capacity of GBM cells increasing tumor malignancy [190,191,192,193,194,195]. GBM cells crosscommunicate with the cells of the microenvironment by secreting a variety of cytokines that promote immunosuppression, increase angiogenesis, and decrease T-cell activity [166,196,197,198,199,200]. They also release chemoattractants, such as CXCL12 [201], CSF-1 [202], and CCL2 [203,204] to recruit myeloid cells [201]. Ultimately, glioma growth occurs in a specialized immunosuppressive microenvironment that promotes proliferation and invasion [190,197].

RelB was identified as a prognostic marker for GBM [108,205,206]. Analysis of patients data from the Cancer Genome Atlas (TCGA) and the Chinese Glioma Genome Atlas (CGGA) indicates that increased RelB expression correlates with more severe glioma grade, shorter life expectancy, and overall negative prognosis [205,206]. High RelB expression associates with programs linked to pathways for both the innate and adaptive immune responses, apoptosis, and cell adhesion [205].

Multiple specific RelB-dependent mechanisms have been proposed to date that function in GBM cells. RelB was shown to drive GBM progression through the induction of the proneural to highly aggressive mesenchymal transition [105,207,208]. This progression may depend on GAMs that release extracellular vesicles with microRNAs (miRNAs) [105]. These miRNAs target chromodomain helicase DNA-binding protein 7 (CDH7), which is upregulated in proneural GBM and prevents progression to the mesenchymal subtype [105,209]. Interestingly, the inhibition of CDH7 activates both the RelB/p50 and the p-STAT3 but is independent of p-p65 [105], although p65 was previously shown to regulate this transition [210]. RelB also promotes the expression of CHI3L1 (YKL40), a marker of the mesenchymal subtype [42,105,206,211]. RelB expression also correlates with the expression of a long-coding RNA, LOXL1-AS1 [207]. Interestingly, decreased LOXL1-AS1 expression limits RelB levels, repressed CD44 mesenchymal subtype marker expression, and induced Olig2 proneural subtype marker abundance [207]. However, overexpression of RelB also induces the expression of Olig2 [206]. Altogether, RelB plays a critical role in tumor progression through the induction of the proneural to mesenchymal transition.

There is substantial evidence implicating the noncanonical pathway in GBM [106,206,212,213,214,215]; however, only some of the effects are likely mediated by RelB. Additionally, activation of the RelB canonical pathway has also been implicated in GBM [105,108]. On this note, aberrant NF-κB signaling through both p65/p50 and RelB/p52 heterodimers correlates with cancer progression (Reviewed by [216]). Smac mimetics were initially identified as small-molecule inhibitors of apoptosis (IAP) antagonist that block antiapoptotic functions [217,218]. However, the Smac mimetic BV6 has broader roles in cancer [215]. BV6 promotes GBM cell migration and invasion through the activation of the noncanonical NF-κB pathway [215,219,220], including binding of RelB, p52, and p50 subunits to the DNA, and the induction of target genes, such as TNFα [215]. BV6 also promotes the differentiation of GBM cancer stem-like cells in a RelB/p52-dependent manner, inducing the expression of CD133, Nanog, and Sox2 as well as GFAP [219]. Furthermore, tumor necrosis factor-like weak inducer of apoptosis (TWEAK), which activates the noncanonical pathway [34,106], induces accumulation of RelB in the nuclei of GBM cells and promotes GBM growth and invasion [106]. RelB has also been shown to promote proliferation of glioma-initiating cells as well as tumor growth and invasion [107,206]. However, even though the noncanonical pathway has been greatly implicated in GBM progression, sulfasalazine, an inhibitor of NF-κB, failed GBM clinical trials [221].

In addition to the noncanonical pathway-activated RelB/p52 signaling, activation of the RelB/p50 complexes by the canonical pathway is also a critical event promoting the immunosuppressive state and GBM progression [108]. High expression levels of both IL-1β and oncostatin M (OSM) correlate with poor patient prognosis. Furthermore, IL-1β and OSM induce RelB/p50 heterodimers formation and their translocation to the nuclei and surprisingly induce expression of proinflammatory cytokines [108]. This directly contrasts the anti-inflammatory role of RelB/p50 heterodimers in astrocytes [47]. Unlike RelB/p50 repression of cytokine genes in astrocytes [47], RelB/p50-mediated activation of cytokines in GBM cells is independent of SIRT1 and likely involves Yin Yang 1 (YY1) [47,79,108]. Interestingly, expression of colony stimulating factor 1 (CSF1), CSF2, CSF3, C-C motif ligand 2 (CCL2), CCL7, and C-X-C motif ligand 2 (CXCL2), which promote myeloid cell recruitment [222] and drive tumor progression [223], was dependent on RelB [108]. Ultimately, it was proposed that RelB acts as a molecular switch in GBM promoting chronic immunosuppressive inflammation [108].

## 5. Conclusions

Activation of the NF-κB pathways and p65 during neuroinflammation is relatively well examined. However, the role of RelB has been overlooked for a long time. Accumulating data indicate that RelB plays critical roles in coordinating neuroinflammatory responses in the CNS. In addition, RelB may be one of the critical factors in the development of the immunosuppressive state associated with GBM.

## Figures and Tables

**Table 1:** Mechanisms of RelB action in the central nervous system (CNS).

Model	Mechanism	Cell Type	Reference
LPS induced tolerance	Epigenetic silencing	Mouse microglia	[99]
HIV/Tat induction	Repression of p65	Mouse microglia	[102]
IL-1β induced tolerance	Dimer switching/phosphorylation	Human astrocytes	[47]
EAE	Repression of p65	Mouse oligodendrocytes	[104]
miRNA inhibition of CDH7	Activation of RelB/p50	Human glioma stem cells	[105]
TWEAK induction	Activation of noncanonical pathway (RelB/p52)	Human glioma cells	[106]
Overexpression of Eva1	Activation of noncanonical pathway (RelB/p52)	Human glioma-initiating cells	[107]
IL-1 β and OSM stimulation	RelB/p50/YY1 complex formation	Human GBM cells	[108]

## Data Availability

Not applicable.

## References

[1] Lauro C., Limatola C. (2020). Metabolic Reprograming of Microglia in the Regulation of the Innate Inflammatory Response. Front. Immunol..

[2] Phatnani H., Maniatis T. (2015). Astrocytes in neurodegenerative disease. Cold Spring Harb. Perspect. Biol..

[3] Kwon H.S., Koh S.H. (2020). Neuroinflammation in neurodegenerative disorders: The roles of microglia and astrocytes. Transl. Neurodegener..

[4] Schonhoff A.M., Williams G.P., Wallen Z.D., Standaert D.G., Harms A.S. (2020). Innate and Adaptive Immune Responses in Parkinson’s Disease.

[5] Netea M.G., Joosten L.A.B., Latz E., Mills K.H.G., Natoli G., Stunnenberg H.G., O’Neill L.A.J., Xavier R.J. (2016). Trained immunity: A program of innate immune memory in health and disease. Science.

[6] Labzin L.I., Heneka M.T., Latz E. (2018). Innate immunity and neurodegeneration. Annu. Rev. Med..

[7] Forrester J.V., McMenamin P.G., Dando S.J. (2018). CNS infection and immune privilege. Nat. Rev. Neurosci..

[8] Linnerbauer M., Wheeler M.A., Quintana F.J. (2020). Astrocyte crosstalk in CNS inflammation. Neuron.

[9] Falcão A.M., van Bruggen D., Marques S., Meijer M., Jäkel S., Agirre E.S., Floriddia E.M., Vanichkina D.P., French-Constant C. (2018). Disease-specific oligodendrocyte lineage cells arise in multiple sclerosis. Nat. Med..

[10] Moyon S., Dubessy A.L., Aigrot M.S., Trotter M., Huang J.K., Dauphinot L., Potier M.C., Kerninon C., Parsadaniantz S.M., Franklin R.J.M. (2015). Demyelination causes adult CNS progenitors to revert to an immature state and express immune cues that support their migration. J. Neurosci..

[11] Zeis T., Enz L., Schaeren-Wiemers N. (2016). The immunomodulatory oligodendrocyte. Brain Res..

[12] Zaman V., Shields D.C., Shams R., Drasites K.P., Matzelle D., Haque A., Banik N.L. (2021). Cellular and molecular pathophysiology in the progression of Parkinson’s disease. Metab. Brain Dis..

[13] Chen W.W., Zhang X., Huang W.J. (2016). Role of neuroinflammation in neurodegenerative diseases (Review). Mol. Med. Rep..

[14] Stephenson J., Nutma E., van der Valk P., Amor S. (2018). Inflammation in CNS neurodegenerative diseases. Immunology.

[15] Ransohoff R.M. (2016). How neuroinflammation contributes to neurodegeneration. Science.

[16] Taylor J.P., Hardy J., Fischbeck K.H. (2002). Toxic proteins in neurodegenerative disease. Science.

[17] Chen H., Chan D.C. (2009). Mitochondrial dynamics-fusion, fission, movement, and mitophagy-in neurodegenerative diseases. Hum. Mol. Genet..

[18] Jellinger K.A. (2010). Basic mechanisms of neurodegeneration: A critical update. J. Cell. Mol. Med..

[19] Redmann M., Darley-Usmar V., Zhang J. (2016). The role of autophagy, mitophagy and lysosomal functions in modulating bioenergetics and survival in the context of redox and proteotoxic damage: Implications for neurodegenerative diseases. Aging Dis..

[20] Shabab T., Khanabdali R., Moghadamtousi S.Z., Kadir H.A., Mohan G. (2017). Neuroinflammation pathways: A general review. Int. J. Neurosci..

[21] Jin W., Qazi T.J., Quan Z., Li N., Qing H. (2019). Dysregulation of Transcription Factors: A Key Culprit Behind Neurodegenerative Disorders. Neuroscientist.

[22] Oeckinghaus A., Ghosh S. (2009). The NF-kappaB family of transcription factors and its regulation. Cold Spring Harb. Perspect. Biol..

[23] Zhou Y., Cui C., Ma X., Luo W., Zheng S.G., Qiu W. (2020). Nuclear Factor κB (NF-κB)–Mediated Inflammation in Multiple Sclerosis. Front. Immunol..

[24] Yang M.G., Sun L., Han J., Zheng C., Liang H., Zhu J., Jin T. (2019). Biological characteristics of transcription factor RelB in different immune cell types: Implications for the treatment of multiple sclerosis. Mol. Brain.

[25] Shih V.F.S., Tsui R., Caldwell A., Hoffmann A. (2011). A single NFκB system for both canonical and non-canonical signaling. Cell Res..

[26] Derudder E., Dejardin E., Pritchard L.L., Green D.R., Körner M., Baud V. (2003). RelB/p50 dimers are differentially regulated by tumor necrosis factor-α and lymphotoxin-β receptor activation. Critical roles for p100. J. Biol. Chem..

[27] Israel A. (2010). The IKK Complex, a Central Regulator of NF- B Activation. Cold Spring Harb. Perspect. Biol..

[28] Senftleben U., Cao Y., Xiao G., Greten F.R., Krähn G., Bonizzi G., Chen Y., Hu Y., Fong A., Sun S.C. (2001). Activation by IKKα of a second, evolutionary conserved, NF-κB signaling pathway. Science.

[29] Hayden M.S., Ghosh S. (2012). NF-κB, the first quarter-century: Remarkable progress and outstanding questions. Genes Dev..

[30] Cildir G., Low K.C., Tergaonkar V. (2016). Noncanonical NF-κB Signaling in Health and Disease. Trends Mol. Med..

[31] Yilmaz Z.B., Weih D.S., Sivakumar V., Weih F. (2003). RelB is required for Peyer’s patch development: Differential regulation of p52-RelB by lymphotoxin and TNF. EMBO J..

[32] Coope H.J., Atkinson P.G.P., Huhse B., Belich M., Janzen J., Holman M.J., Klaus G.G.B., Johnston L.H., Ley S.C. (2002). CD40 regulates the processing of NF-κB2 p100 to p52. EMBO J..

[33] Claudio E., Brown K., Park S., Wang H., Siebenlist U. (2002). BAFF-induced NEMO-independent processing of NF-κB2 in maturing B cells. Nat. Immunol..

[34] Saitoh T., Nakayama M., Nakano H., Yagita H., Yamamoto N., Yamaoka S. (2003). TWEAK Induces NF-κB2 p100 processing and long lasting nf-κB Activation. J. Biol. Chem..

[35] Vince J.E., Wong W.W.L., Khan N., Feltham R., Chau D., Ahmed A.U., Benetatos C.A., Chunduru S.K., Condon S.M., McKinlay M. (2007). IAP antagonists target cIAP1 to Induce TNFα-dependent apoptosis. Cell.

[36] Gardam S., Sierro F., Basten A., Mackay F., Brink R. (2008). TRAF2 and TRAF3 signal adapters act cooperatively to control the maturation and survival signals delivered to B Cells by the BAFF Receptor. Immunity.

[37] Vallabhapurapu S., Matsuzawa A., Zhang W.Z., Tseng P.H., Keats J.J., Wang H., Vignali D.A.A., Bergsagel P.L., Karin M. (2008). Nonredundant and complementary functions of TRAF2 and TRAF3 in a ubiquitination cascade that activates NIK-dependent alternative NF-κB signaling. Nat. Immunol..

[38] Xiao G., Harhaj E.W., Sun S.C. (2001). NF-κB-Inducing kinase regulates the processing of NF-κB2 p100. Mol. Cell.

[39] Fong A., Sun S.C. (2002). Genetic evidence for the essential role of β-transducin repeat-containing protein in the inducible processing of NF-κB2/p100. J. Biol. Chem..

[40] Millet P., McCall C., Yoza B. (2013). RelB: An outlier in leukocyte biology. J. Leukoc. Biol..

[41] Gasparini C., Foxwell B.M.J., Feldmann M. (2009). RelB/p50 regulates CCL19 production, but fails to promote human DC maturation. Eur. J. Immunol..

[42] Bhardwaj R., Yester J.W., Singh S.K., Biswas D.D., Surace M.J., Waters M.R., Hauser K.F., Yao Z., Boyce B.F., Kordula T. (2015). RelB/p50 Complexes Regulate Cytokine-Induced YKL-40 Expression. J. Immunol..

[43] Shih V.F.S., Davis-Turak J., MacAl M., Huang J.Q., Ponomarenko J., Kearns J.D., Yu T., Fagerlund R., Asagiri M., Zuniga E.I. (2012). Control of RelB during dendritic cell activation integrates canonical and noncanonical NF-κB pathways. Nat. Immunol..

[44] Solan N.J., Miyoshi H., Carmona E.M., Bren G.D., Paya C.V. (2002). RelB cellular regulation and transcriptional activity are regulated by p100. J. Biol. Chem..

[45] Fusco A.J., Savinova O.V., Talwar R., Kearns J.D., Hoffmann A., Ghosh G. (2008). Stabilization of RelB requires multidomain interactions with p100/p52. J. Biol. Chem..

[46] Carrasco D., Ryseck R.P., Bravo R. (1993). Expression of relB transcripts during lymphoid organ development: Specific expression in dendritic antigen-presenting cells. Development.

[47] Gupta A.S., Waters M.R., Biswas D.D., Brown L.N., Surace M.J., Floros C., Siebenlist U., Kordula T. (2019). RelB controls adaptive responses of astrocytes during sterile inflammation. Glia.

[48] Chen X., Yoza B.K., El Gazzar M., Hu J.Y.Q., Cousart S.L., McCall C.E. (2009). RelB Sustains IκBα Expression during Endotoxin Tolerance. Clin. Vaccine Immunol..

[49] Chen X., El Gazzar M., Yoza B.K., McCall C.E. (2009). The NF-κB factor RelB and histone H3 lysine methyltransferase G9a directly interact to generate epigenetic silencing in endotoxin tolerance. J. Biol. Chem..

[50] Marienfeld R., May M.J., Berberich I., Serfling E., Ghosh S., Neumann M. (2003). RelB forms transcriptionally inactive complexes with RelA/p65. J. Biol. Chem..

[51] RELB RELB proto-oncogene, NF-kB subunit [ Homo sapiens (human). https://www.ncbi.nlm.nih.gov/gene/5971.

[52] Bren G.D., Solan N.J., Miyoshi H., Pennington K.N., Pobst L.J., Paya C.V. (2001). Transcription of the RelB gene is regulated by NF-κB. Oncogene.

[53] Lernbecher T., Kistler B., Wirth T. (1994). Two distinct mechanisms contribute to the constitutive activation of RelB in lymphoid cells. EMBO J..

[54] Ammon C., Mondal K., Andreesen R., Krause S.W. (2000). Differential expression of the transcription factor NF-κB during human mononuclear phagocyte differentiation to macrophages and dendritic cells. Biochem. Biophys. Res. Commun..

[55] Ruben S.M., Klement J.F., Coleman T.A., Maher M., Chen C.H., Rosen C.A. (1992). I-Rel: A novel rel-related protein that inhibits NF-κB transcriptional activity. Genes Dev..

[56] Cramer P., Müller C.W. (1997). Engineering of diffraction-quality crystals of the NF-κB p52 homodimer:DNA complex. FEBS Lett..

[57] Ghosh G., Van Duyne G., Ghosh S., Sigler P.B. (1995). Structure of nf-κb p50 homodimer bound to a κb site. Nature.

[58] Müller C.W., Rey F.A., Sodeoka M., Verdine G.L., Harrison S.C. (1995). Structure of the nf-κb p50 homodimer bound to DNA. Nature.

[59] Chen F.E., Huang D.B., Chen Y.Q., Ghosh G. (1998). Crystal structure of p50/p65 heterodimer of transcription factor NF-κb bound to DNA. Nature.

[60] Chen Y.-Q., Ghosh S., Ghosh G. (1998). A novel DNA recognition mode by the NF-κB p65 homodimer. Nat. Struct. Biol..

[61] Huang D.B., Chen Y.Q., Ruetsche M., Phelps C.B., Ghosh G. (2001). X-ray crystal structure of proto-oncogene product c-Rel bound to the CD28 response element of IL-2. Structure.

[62] Ryseck R.P., Novotny J., Bravo R. (1995). Characterization of elements determining the dimerization properties of RelB and p50. Mol. Cell. Biol..

[63] Maier H.J., Marienfeld R., Wirth T., Baumann B. (2003). Critical role of RelB serine 368 for dimerization and p100 stabilization. J. Biol. Chem..

[64] Ryseck R.P., Bull P., Takamiya M., Bours V., Siebenlist U., Dobrzanski P., Bravo R. (1992). RelB, a new Rel family transcription activator that can interact with p50-NF-kappa B. Mol. Cell. Biol..

[65] May M.J., Ghosh S. (1997). Rel/NF-κB and IκB proteins: An overview. Semin. Cancer Biol..

[66] Moorthy A.K., Huang D.B., Wang V.Y.F., Vu D., Ghosh G. (2007). X-ray Structure of a NF-κB p50/RelB/DNA complex reveals assembly of multiple dimers on tandem κB Sites. J. Mol. Biol..

[67] Dobrzanski P., Ryseck R.P., Bravo R. (1993). Both N- and C-terminal domains of RelB are required for full transactivation: Role of the N-terminal leucine zipper-like motif. Mol. Cell. Biol..

[68] Baud V., Collares D. (2016). Post-translational modifications of RelB NF-κB subunit and associated functions. Cells.

[69] Marienfeld R., Berberich-Siebelt F., Berberich I., Denk A., Serfling E., Neumann M. (2001). Signal-specific and phosphorylation-dependent RelB degradation: A potential mechanism of NF-κB control. Oncogene.

[70] Neumann M., Klar S., Wilisch-Neumann A., Hollenbach E., Kavuri S., Leverkus M., Kandolf R., Brunner-Weinzierl M.C., Klingel K. (2011). Glycogen synthase kinase-3Β is a crucial mediator of signal-induced RelB degradation. Oncogene.

[71] Authier H., Billot K., Derudder E., Bordereaux D., Riviere P., Rodrigues-Ferreira S., Nahmias C., Baud V. (2014). IKK phosphorylates RelB to modulate its promoter specificity and promote fibroblast migration downstream of TNF receptors. Proc. Natl. Acad. Sci. USA.

[72] Weih D.S., Yilmaz Z.B., Weih F. (2001). Essential Role of RelB in germinal center and marginal zone formation and proper expression of homing chemokines. J. Immunol..

[73] Gerondakis S., Grumont R., Gugasyan R., Wong L., Isomura I., Ho W., Banerjee A. (2006). Unravelling the complexities of the NF-κB signalling pathway using mouse knockout and transgenic models. Oncogene.

[74] Burkly L., Hession C., Ogata L., Reilly C., Marconl L.A., Olson D., Tizard R., Gate R., Lo D. (1995). Expression of relB is required for the development of thymic medulla and dendritic cells. Nature.

[75] Vaira S., Johnson T., Hirbe A.C., Alhawagri M., Anwisye I., Sammut B., O’Neal J., Zou W., Weilbaecher K.N., Faccio R. (2008). RelB is the NF-κB subunit downstream of NIK responsible for osteoclast differentiation. Proc. Natl. Acad. Sci. USA.

[76] Sun S.-C. (2012). The noncanonical NF-κB pathway. Immunol. Rev..

[77] Weih F., Durham S.K., Barton D.S., Sha W.C., Baltimore D., Bravo R. (1996). Both multiorgan inflammation and myeloid hyperplasia in RelB-deficient mice are T cell dependent. J. Immunol..

[78] Yoza B.K., Hu J.Y.-Q., Cousart S.L., Forrest L.M., McCall C.E. (2006). Induction of RelB participates in endotoxin tolerance. J. Immunol..

[79] Liu T.F., Yoza B.K., El Gazzar M., Vachharajani V.T., McCall C.E. (2011). NAD+-dependent SIRT1 deacetylase participates in epigenetic reprogramming during endotoxin tolerance. J. Biol. Chem..

[80] Zhao X., Zhang Y., Strong R., Zhang J., Grotta J.C., Aronowski J. (2007). Distinct patterns of intracerebral hemorrhage-induced alterations in NF-κB subunit, iNOS, and COX-2 expression. J. Neurochem..

[81] Richa R., Yadawa A.K., Chaturvedi C.M. (2017). Hyperglycemia and high nitric oxide level induced oxidative stress in the brain and molecular alteration in the neurons and glial cells of laboratory mouse, Mus musculus. Neurochem. Int..

[82] Mitra S., Ghosh N., Sinha P., Chakrabarti N., Bhattacharyya A. (2016). Alteration of nuclear factor-kappaB pathway promote neuroinflammation depending on the functions of estrogen receptors in substantia nigra after 1-methyl-4-phenyl-1,2,3,6-tetrahydropyridine treatment. Neurosci. Lett..

[83] Riphagen J.M., Ramakers I.H.G.M., Freeze W.M., Pagen L.H.G., Hanseeuw B.J., Verbeek M.M., Verhey F.R.J., Jacobs H.I.L. (2020). Linking APOE-ε4, blood-brain barrier dysfunction, and inflammation to Alzheimer’s pathology. Neurobiol. Aging.

[84] Bekris L.M., Yu C.-E., Bird T.D., Tsuang D.W. (2010). Review Article: Genetics of Alzheimer Disease. J. Geriatr. Psychiatry Neurol..

[85] Nho K., Kim S., Horgusluoglu E., Risacher S.L., Shen L., Kim D., Lee S., Foroud T., Shaw L.M., Trojanowski J.Q. (2017). Association analysis of rare variants near the APOE region with CSF and neuroimaging biomarkers of Alzheimer’s disease. BMC Med. Genom..

[86] Xiao E., Chen Q., Goldman A.L., Tan H.Y., Healy K., Zoltick B., Das S., Kolachana B., Callicott J.H., Dickinson D. (2017). Late-Onset Alzheimer’s Disease polygenic risk profile score predicts hippocampal function. Biol. Psychiatry Cogn. Neurosci. Neuroimaging.

[87] Yin J., Valin K.L., Dixon M.L., Leavenworth J.W. (2017). The role of microglia and macrophages in CNS homeostasis, autoimmunity, and cancer. J. Immunol. Res..

[88] Mosser C.A., Baptista S., Arnoux I., Audinat E. (2017). Microglia in CNS development: Shaping the brain for the future. Prog. Neurobiol..

[89] Ginhoux F., Lim S., Hoeffel G., Low D., Huber T. (2013). Origin and differentiation of microglia. Front. Cell. Neurosci..

[90] Nimmerjahn A., Kirchhoff F., Helmchen F. (2005). Resting microglial cells are highly dynamic surveillants of brain parenchyma in vivo. Science.

[91] Kettenmann H., Hanisch U.K., Noda M., Verkhratsky A. (2011). Physiology of microglia. Physiol. Rev..

[92] Xu Y., Jin M.Z., Yang Z.Y., Jin W.L. (2021). Microglia in neurodegenerative diseases. Neural Regen. Res..

[93] Orihuela R., McPherson C.A., Harry G.J. (2016). Microglial M1/M2 polarization and metabolic states. Br. J. Pharmacol..

[94] Kabba J.A., Xu Y., Christian H., Ruan W., Chenai K., Xiang Y., Zhang L., Saavedra J.M., Pang T. (2018). Microglia: Housekeeper of the central nervous system. Cell. Mol. Neurobiol..

[95] Neumann H., Kotter M.R., Franklin R.J.M. (2009). Debris clearance by microglia: An essential link between degeneration and regeneration. Brain.

[96] Foster S.L., Hargreaves D.C., Medzhitov R. (2007). Gene-specific control of inflammation by TLR-induced chromatin modifications. Nature.

[97] Netea M.G., Domínguez-Andrés J., Barreiro L.B., Chavakis T., Divangahi M., Fuchs E., Joosten L.A.B., van der Meer J.W.M., Mhlanga M.M., Mulder W.J.M. (2020). Defining trained immunity and its role in health and disease. Nat. Rev. Immunol..

[98] Wendeln A.-C., Degenhardt K., Kaurani L., Gertig M., Ulas T., Jain G., Wagner J., Häsler L.M., Wild K., Skodras A. (2018). Innate immune memory in the brain shapes neurological disease hallmarks. Nature.

[99] Schaafsma W., Zhang X., van Zomeren K.C., Jacobs S., Georgieva P.B., Wolf S.A., Kettenmann H., Janova H., Saiepour N., Hanisch U.K. (2015). Long-lasting pro-inflammatory suppression of microglia by LPS-preconditioning is mediated by RelB-dependent epigenetic silencing. Brain. Behav. Immun..

[100] Tay T.L., Mai D., Dautzenberg J., Fernández-Klett F., Lin G., Sagar S., Datta M., Drougard A., Stempfl T., Ardura-Fabregat A. (2017). A new fate mapping system reveals context-dependent random or clonal expansion of microglia. Nat. Neurosci..

[101] Füger P., Hefendehl J.K., Veeraraghavalu K., Wendeln A.C., Schlosser C., Obermüller U., Wegenast-Braun B.M., Neher J.J., Martus P., Kohsaka S. (2017). Microglia turnover with aging and in an Alzheimer’s model via long-term in vivo single-cell imaging. Nat. Neurosci..

[102] Kiebala M., Polesskaya O., Yao Z., Perry S.W., Maggirwa S.B. (2010). Nuclear factor-kappa B family member RelB inhibits human immunodeficiency virus-1 Tat-induced tumor necrosis factor-alpha production. PLoS ONE.

[103] Laspia M.F., Rice A.P., Mathews M.B. (1989). HIV-1 Tat protein increases transcriptional initiation and stabilizes elongation. Cell.

[104] Gupta A.S., Biswas D.D., Brown L.S.N., Mockenhaupt K., Marone M., Hoskins A., Siebenlist U., Kordula T. (2019). A detrimental role of RelB in mature oligodendrocytes during experimental acute encephalomyelitis. J. Neuroinflammation.

[105] Zhang Z., Xu J., Chen Z., Wang H., Xue H., Yang C., Guo Q., Qi Y., Guo X., Qian M. (2020). Transfer of MicroRNA via macrophage-derived extracellular vesicles promotes proneural-to-mesenchymal transition in glioma stem cells. Cancer Immunol. Res..

[106] Cherry E.M., Lee D.W., Jung J.-U., Sitcheran R. (2015). Tumor necrosis factor-like weak inducer of apoptosis (TWEAK) promotes glioma cell invasion through induction of NF-κB-inducing kinase (NIK) and noncanonical NF-κB signaling. Mol. Cancer.

[107] Ohtsu N., Nakatani Y., Yamashita D., Ohue S., Ohnishi T., Kondo T. (2016). Eva1 maintains the stem-like character of glioblastoma-initiating cells by activating the noncanonical NF-κB signaling pathway. Cancer Res..

[108] Waters M.R., Gupta A.S., Mockenhaupt K., Brown L.S.N., Biswas D.D., Kordula T. (2019). RelB acts as a molecular switch driving chronic inflammation in glioblastoma multiforme. Oncogenesis.

[109] Song S., Luo L., Sun B., Sun D. (2020). Roles of glial ion transporters in brain diseases. Glia.

[110] Zuchero J.B., Barres B.A. (2015). Glia in mammalian development and disease. Development.

[111] Zhang Y., Barres B.A. (2010). Astrocyte heterogeneity: An underappreciated topic in neurobiology. Curr. Opin. Neurobiol..

[112] Khakh B.S., Sofroniew M.V. (2015). Diversity of astrocyte functions and phenotypes in neural circuits. Nat. Neurosci..

[113] Farmer W.T., Murai K. (2017). Resolving Astrocyte Heterogeneity in the CNS. Front. Cell. Neurosci..

[114] Sofroniew M.V. (2009). Molecular dissection of reactive astrogliosis and glial scar formation. Trends Neurosci..

[115] Jang E., Kim J.-H., Lee S., Kim J.-H., Seo J.-W., Jin M., Lee M.-G., Jang I.-S., Lee W.-H., Suk K. (2013). Phenotypic Polarization of Activated Astrocytes: The Critical Role of Lipocalin-2 in the Classical Inflammatory Activation of Astrocytes. J. Immunol..

[116] Beurel E. (2011). HDAC6 regulates LPS-tolerance in astrocytes. PLoS ONE.

[117] Saccani S., Pantano S., Natoli G. (2003). Modulation of NF-κB activity by exchange of dimers. Mol. Cell.

[118] Shih V.F.S., Kearns J.D., Basak S., Savinova O.V., Ghosh G., Hoffmann A. (2009). Kinetic control of negative feedback regulators of NF-κB/RelA determines their pathogen- and cytokine-receptor signaling specificity. Proc. Natl. Acad. Sci. USA.

[119] Yeung F., Hoberg J.E., Ramsey C.S., Keller M.D., Jones D.R., Frye R.A., Mayo M.W. (2004). Modulation of NF-κB-dependent transcription and cell survival by the SIRT1 deacetylase. EMBO J..

[120] Mayo L., Trauger S.A., Blain M., Nadeau M., Patel B., Alvarez J.I., Mascanfroni I.D., Yeste A., Kivisäkk P., Kallas K. (2014). Regulation of astrocyte activation by glycolipids drives chronic CNS inflammation. Nat. Med..

[121] Swindell W.R., Bojanowski K., Chaudhuri R.K. (2020). A novel fumarate, isosorbide di-(methyl fumarate) (IDMF), replicates astrocyte transcriptome responses to dimethyl fumarate (DMF) but specifically down-regulates genes linked to a reactive phenotype. Biochem. Biophys. Res. Commun..

[122] Bradl M., Lassmann H. (2010). Oligodendrocytes: Biology and pathology. Acta Neuropathol..

[123] Kuhn S., Gritti L., Crooks D., Dombrowski Y. (2019). Oligodendrocytes in Development, Myelin Generation and Beyond. Cells.

[124] Wilkins A., Majed H., Layfield R., Compston A., Chandran S. (2003). Oligodendrocytes promote neuronal survival and axonal length by distinct intracellular mechanisms: A novel role for oligodendrocyte-derived glial cell line-derived neurotrophic factor. J. Neurosci..

[125] Tiane A., Schepers M., Rombaut B., Hupperts R., Prickaerts J., Hellings N., van den Hove D., Vanmierlo T. (2019). From OPC to Oligodendrocyte: An epigenetic journey. Cells.

[126] Gibson E.M., Purger D., Mount C.W., Goldstein A.K., Lin G.L., Wood L.S., Inema I., Miller S.E., Bieri G., Zuchero J.B. (2014). Neuronal activity promotes oligodendrogenesis and adaptive myelination in the mammalian brain. Science.

[127] Dowling P., Husar W., Menonna J., Donnenfeld H., Cook S., Sidhu M. (1997). Cell death and birth in multiple sclerosis brain. J. Neurol. Sci..

[128] Pender M.P., Nguyen K.B., McCombe P.A., Kerr J.F.R. (1991). Apoptosis in the nervous system in experimental allergic encephalomyelitis. J. Neurol. Sci..

[129] Hisahara S., Okano H., Miura M. (2003). Caspase-mediated oligodendrocyte cell death in the pathogenesis of autoimmune demyelination. Neurosci. Res..

[130] Schmitz T., Chew L.J. (2008). Cytokines and myelination in the central nervous system. Sci. World J..

[131] Nicholas R.S.T.J., Wing M.G., Compston A. (2001). Nonactivated microglia promote oligodendrocyte precursor survival and maturation through the transcription factor NF-κB. Eur. J. Neurosci..

[132] Hamanoue M., Yoshioka A., Ohashi T., Eto Y., Takamatsu K. (2004). NF-kappaB prevents TNF-alpha-induced apoptosis in an oligodendrocyte cell line. Neurochem. Res..

[133] Stone S., Jamison S., Yue Y., Durose W., Schmidt-Ullrich R., Lin W. (2017). NF-κB activation protects oligodendrocytes against inflammation. J. Neurosci..

[134] Lei Z., Yue Y., Stone S., Wu S., Lin W. (2020). NF-κB activation accounts for the cytoprotective effects of PERK activation on oligodendrocytes during EAE. J. Neurosci..

[135] Karin M., Lin A. (2002). NF-κB at the crossroads of life and death. Nat. Immunol..

[136] Chu Z.-L., McKinsey T.A., Liu L., Gentry J.J., Malim M.H., Ballard D.W. (1997). Suppression of tumor necrosis factor-induced cell death by inhibitor of apoptosis c-IAP2 is under NF- B control. Proc. Natl. Acad. Sci. USA.

[137] Tsunemoto R., Lee S., Szűcs A., Chubukov P., Sokolova I., Blanchard J.W., Eade K.T., Bruggemann J., Wu C., Torkamani A. (2018). Diverse reprogramming codes for neuronal identity. Nature.

[138] Zhang R.S., Liakath-Ali K., Südhof T.C. (2020). Latrophilin-2 and latrophilin-3 are redundantly essential for parallel-fiber synapse function in cerebellum. Elife.

[139] Hassan B.A., Hiesinger P.R. (2015). Beyond Molecular Codes: Simple Rules to Wire Complex Brains. Cell.

[140] Yogev S., Shen K. (2014). Cellular and Molecular Mechanisms of Synaptic Specificity. Annu. Rev. Cell Dev. Biol..

[141] Li H., Shuster S.A., Li J., Luo L. (2018). Linking neuronal lineage and wiring specificity. Neural Dev..

[142] Engelmann C., Haenold R. (2016). Transcriptional Control of Synaptic Plasticity by Transcription Factor NF-κB. Neural Plast..

[143] Dresselhaus E.C., Meffert M.K. (2019). Cellular specificity of NF-κB function in the nervous system. Front. Immunol..

[144] Engelmann C., Riemann M., Carlstedt S., Grimlowski R., Andreas N., Koliesnik I., Meier E., Austerfield P., Haenold R. (2020). Identification of undescribed Relb expression domains in the murine brain by new Relb:cre-katushka reporter mice. Dev. Dyn..

[145] Schmeisser M.J., Baumann B., Johannsen S., Vindedal G.F., Jensen V., Hvalby O.C., Sprengel R., Seither J., Maqbool A., Magnutzki A. (2012). IκB kinase/nuclear factor κB-dependent insulin-like growth factor 2 (Igf2) expression regulates synapse formation and spine maturation via Igf2 receptor signaling. J. Neurosci..

[146] Bhakar A.L., Tannis L.L., Zeindler C., Russo M.P., Jobin C., Park D.S., MacPherson S., Barker P.A. (2002). Constitutive nuclear factor-κB activity is required for central neuron survival. J. Neurosci..

[147] Sun Y., Huang X., Liu M., Cao J., Chen J., Wang H., Niu H., Yu Z., Yu J., Wang T. (2012). A new alternative NF-ΚB Pathway mediated the neuroprotection of GDNF on 6-OHDA-induced da neurons neurotoxicity. Brain Res..

[148] Xiao X., Putatunda R., Zhang Y., Soni P.V., Li F., Zhang T., Xin M., Luo J.J., Bethea J.R., Cheng Y. (2018). Lymphotoxin β receptor-mediated NFκB signaling promotes glial lineage differentiation and inhibits neuronal lineage differentiation in mouse brain neural stem/progenitor cells. J. Neuroinflammation.

[149] Calandria J.M., Asatryan A., Balaszczuk V., Knott E.J., Jun B.K., Mukherjee P.K., Belayev L., Bazan N.G. (2015). NPD1-mediated stereoselective regulation of BIRC3 expression through cREL is decisive for neural cell survival. Cell Death Differ..

[150] Smyth L.C.D., Rustenhoven J., Park T.I.H., Schweder P., Jansson D., Heppner P.A., O’Carroll S.J., Mee E.W., Faull R.L.M., Curtis M. (2018). Unique and shared inflammatory profiles of human brain endothelia and pericytes. J. Neuroinflammation.

[151] Rudziak P., Ellis C.G., Kowalewska P.M. (2019). Role and Molecular Mechanisms of Pericytes in Regulation of Leukocyte Diapedesis in Inflamed Tissues. Mediators Inflamm..

[152] Qin W., Li J., Zhu R., Gao S., Fan J., Xia M., Zhao R.C., Zhang J. (2019). Melatonin protects blood-brain barrier integrity and permeability by inhibiting matrix metalloproteinase-9 via the NOTCH3/NF-kB pathway. Aging.

[153] Yun J.-H. (2021). Interleukin-1β induces pericyte apoptosis via the NF-κB pathway in diabetic retinopathy. Biochem. Biophys. Res. Commun..

[154] Liang Q., Zhang L., Wood R.W., Ji R.C., Boyce B.F., Schwarz E.M., Wang Y., Xing L. (2019). Avian reticuloendotheliosis viral oncogene related B regulates lymphatic endothelial cells during vessel maturation and is required for lymphatic vessel function in adult mice. Am. J. Pathol..

[155] Andreas N., Potthast M., Geiselhöringer A.-L., Garg G., de Jong R., Riewaldt J., Russkamp D., Riemann M., Girard J.-P., Blank S. (2019). RelB deficiency in dendritic cells protects from autoimmune inflammation due to spontaneous accumulation of Tissue T regulatory cells. J. Immunol..

[156] Obara-Michlewska M., Szeliga M. (2020). Targeting glutamine addiction in Gliomas. Cancers.

[157] Caniglia J.L., Jalasutram A., Asuthkar S., Sahagun J., Park S., Ravindra A., Tsung A.J., Guda M.R., Velpula K.K. (2021). Beyond glucose: Alternative sources of energy in glioblastoma. Theranostics.

[158] Russo C., Lisi L., Tentori L., Navarra P., Graziani G., Combs C. (2017). Exploiting microglial functions for the treatment of glioblastoma. Curr. Cancer Drug Targets.

[159] Tykocki T., Eltayeb M. (2018). Ten-year survival in glioblastoma. A systematic review. J. Clin. Neurosci..

[160] Omuro A., DeAngelis L.M. (2013). Glioblastoma and other malignant gliomas: A clinical review. J. Am. Med. Assoc..

[161] Tanaka S., Louis D.N., Curry W.T., Batchelor T.T., Dietrich J. (2013). Diagnostic and therapeutic avenues for glioblastoma: No longer a dead end?. Nat. Rev. Clin. Oncol..

[162] Behnan J., Finocchiaro G., Hanna G. (2019). The landscape of the mesenchymal signature in brain tumours. Brain.

[163] Wang Q., Hu B., Hu X., Kim H., Squatrito M., Scarpace L., deCarvalho A.C., Lyu S., Li P., Li Y. (2017). Tumor evolution of glioma-intrinsic gene expression subtypes associates with immunological changes in the microenvironment. Cancer Cell.

[164] Behnan J., Stangeland B., Hosainey S.A.M., Joel M., Olsen T.K., Micci F., Glover J.C., Isakson P., Brinchmann J.E. (2017). Differential propagation of stroma and cancer stem cells dictates tumorigenesis and multipotency. Oncogene.

[165] Sarkaria J.N., Hu L.S., Parney I.F., Pafundi D.H., Brinkmann D.H., Laack N.N., Giannini C., Burns T.C., Kizilbash S.H., Laramy J.K. (2018). Is the blood-brain barrier really disrupted in all glioblastomas? A critical assessment of existing clinical data. Neuro. Oncol..

[166] Cui X., Morales R.-T.T., Qian W., Wang H., Gagner J.-P., Dolgalev I., Placantonakis D., Zagzag D., Cimmino L., Snuderl M. (2018). Hacking macrophage-associated immunosuppression for regulating glioblastoma angiogenesis. Biomaterials.

[167] Cheng W., Ren X., Zhang C., Cai J., Liu Y., Han S., Wu A. (2016). Bioinformatic profiling identifies an immune-related risk signature for glioblastoma. Neurology.

[168] McLendon R., Friedman A., Bigner D., Van Meir E.G., Brat D.J., Mastrogianakis G.M., Olson J.J., Mikkelsen T., Lehman N., Aldape K. (2008). Comprehensive genomic characterization defines human glioblastoma genes and core pathways. Nature.

[169] Parsons D.W., Jones S., Zhang X., Lin J.C.H., Leary R.J., Angenendt P., Mankoo P., Carter H., Siu I.M., Gallia G.L. (2008). An integrated genomic analysis of human glioblastoma multiforme. Science.

[170] Phillips H.S., Kharbanda S., Chen R., Forrest W.F., Soriano R.H., Wu T.D., Misra A., Nigro J.M., Colman H., Soroceanu L. (2006). Molecular subclasses of high-grade glioma predict prognosis, delineate a pattern of disease progression, and resemble stages in neurogenesis. Cancer Cell.

[171] Verhaak R.G.W., Hoadley K.A., Purdom E., Wang V., Qi Y., Wilkerson M.D., Miller C.R., Ding L., Golub T., Mesirov J.P. (2010). Integrated Genomic Analysis Identifies Clinically Relevant Subtypes of Glioblastoma Characterized by Abnormalities in PDGFRA, IDH1, EGFR, and NF1. Cancer Cell.

[172] Gill B.J., Pisapia D.J., Malone H.R., Goldstein H., Lei L., Sonabend A., Yun J., Samanamud J., Sims J.S., Banu M. (2014). MRI-localized biopsies reveal subtype-specific differences in molecular and cellular composition at the margins of glioblastoma. Proc. Natl. Acad. Sci. USA.

[173] Verhaak R.G.W. (2016). Moving the needle: Optimizing classification for glioma. Sci. Transl. Med..

[174] Liu C., Sage J.C., Miller M.R., Verhaak R.G.W., Hippenmeyer S., Vogel H., Foreman O., Bronson R.T., Nishiyama A., Luo L. (2011). Mosaic analysis with double markers reveals tumor cell of origin in glioma. Cell.

[175] Persson A.I., Petritsch C., Swartling F.J., Itsara M., Sim F.J., Auvergne R., Goldenberg D.D., Vandenberg S.R., Nguyen K.N., Yakovenko S. (2010). Non-stem cell origin for oligodendroglioma. Cancer Cell.

[176] Ledur P.F., Liu C., He H., Harris A.R., Minussi D.C., Zhou H.Y., Shaffrey M.E., Asthagiri A., Lopes M.B.S., Schiff D. (2016). Culture conditions tailored to the cell of origin are critical for maintaining native properties and tumorigenicity of glioma cells. Neuro. Oncol..

[177] Sutcliffe M.D., Galvao R.P., Wang L., Kim J., Rosenfeld L.K., Singh S., Zong H., Janes K.A. (2021). Premalignant oligodendrocyte precursor cells stall in a heterogeneous state of replication stress prior to gliomagenesis. Cancer Res..

[178] Martinez-Lage M., Lynch T.M., Bi Y., Cocito C., Way G.P., Pal S., Haller J., Yan R.E., Ziober A., Nguyen A. (2019). Immune landscapes associated with different glioblastoma molecular subtypes. Acta Neuropathol. Commun..

[179] Wang L., Babikir H., Müller S., Yagnik G., Shamardani K., Catalan F., Kohanbash G., Alvarado B., Di Lullo E., Kriegstein A. (2019). The phenotypes of proliferating glioblastoma cells reside on a single axis of variation. Cancer Discov..

[180] Wurdinger T., Deumelandt K., van der Vliet H.J., Wesseling P., de Gruijl T.D. (2014). Mechanisms of intimate and long-distance cross-talk between glioma and myeloid cells: How to break a vicious cycle. Biochim. Biophys. Acta.

[181] Glass R., Synowitz M. (2014). CNS macrophages and peripheral myeloid cells in brain tumours. Acta Neuropathol..

[182] Poon C.C., Sarkar S., Yong V.W., Kelly J.J.P. (2017). Glioblastoma-associated microglia and macrophages: Targets for therapies to improve prognosis. Brain.

[183] Osterberg N., Ferrara N., Vacher J., Gaedicke S., Niedermann G., Weyerbrock A., Doostkam S., Schaefer H.E., Plate K.H., Machein M.R. (2016). Decrease of VEGF-A in myeloid cells attenuates glioma progression and prolongs survival in an experimental glioma model. Neuro. Oncol..

[184] Hu F., Ku M.C., Markovic D., Dzaye O.D., Lehnardt S., Synowitz M., Wolf S.A., Kettenmann H. (2014). Glioma-associated microglial MMP9 expression is upregulated by TLR2 signaling and sensitive to minocycline. Int. J. Cancer.

[185] Langenfurth A., Rinnenthal J.L., Vinnakota K., Prinz V., Carlo A.S., Stadelmann C., Siffrin V., Peaschke S., Endres M., Heppner F. (2014). Membrane-type 1 metalloproteinase is upregulated in microglia/brain macrophages in neurodegenerative and neuroinflammatory diseases. J. Neurosci. Res..

[186] Gutmann D.H. (2015). Microglia in the tumor microenvironment: Taking their TOLL on glioma biology. Neuro. Oncol..

[187] Han J., Chen X., Chu J., Xu B., Meisen W.H., Chen L., Zhang L., Zhang J., He X., Wang Q.E. (2015). TGFβ treatment enhances glioblastoma virotherapy by inhibiting the innate immune response. Cancer Res..

[188] Fecci P.E., Mitchell D.A., Whitesides J.F., Xie W., Friedman A.H., Archer G.E., Herndon J.E., Bigner D.D., Dranoff G., Sampson J.H. (2006). Increased Regulatory T-Cell Fraction Amidst a Diminished CD4 Compartment Explains Cellular Immune Defects in Patients with Malignant Glioma. Cancer Res..

[189] El Andaloussi A., Lesniak M.S. (2006). An increase in CD4+CD25+FOXP3+ regulatory T cells in tumor-infiltrating lymphocytes of human glioblastoma multiforme. Neuro. Oncol..

[190] Guan X., Hasan M.N., Maniar S., Jia W., Sun D. (2018). Reactive Astrocytes in Glioblastoma Multiforme. Mol. Neurobiol..

[191] Brandao M., Simon T., Critchley G., Giamas G. (2019). Astrocytes, the rising stars of the glioblastoma microenvironment. Glia.

[192] Piperi C., Papavassiliou K.A., Papavassiliou A.G. (2019). Pivotal Role of STAT3 in Shaping Glioblastoma Immune Microenvironment. Cells.

[193] Jahani-As A., Yin H., Soleimani V.D., Haque T., Luchman H.A., Chang N.C., Sincennes M.C., Puram S.V., Scott A.M., Lorimer I.A.J. (2016). Control of glioblastoma tumorigenesis by feed-forward cytokine signaling. Nat. Neurosci..

[194] Priego N., Zhu L., Monteiro C., Mulders M., Wasilewski D., Bindeman W., Doglio L., Martínez L., Martínez-Saez E., Cajal S.R.Y. (2018). STAT3 labels a subpopulation of reactive astrocytes required for brain metastasis article. Nat. Med..

[195] Priego N., Valiente M. (2019). The potential of astrocytes as immune modulators in brain tumors. Front. Immunol..

[196] Fu W., Wang W., Li H., Jiao Y., Huo R., Yan Z., Wang J., Wang S., Wang J., Chen D. (2020). Single-cell atlas reveals complexity of the immunosuppressive microenvironment of initial and recurrent glioblastoma. Front. Immunol..

[197] Grabowski M.M., Sankey E.W., Ryan K.J., Chongsathidkiet P., Lorrey S.J., Wilkinson D.S., Fecci P.E. (2021). Immune suppression in gliomas. J. Neurooncol..

[198] Kennedy B.C., Maier L.M., D’Amico R., Mandigo C.E., Fontana E.J., Waziri A., Assanah M.C., Canoll P., Anderson R.C.E., Anderson D.E. (2009). Dynamics of central and peripheral immunomodulation in a murine glioma model. BMC Immunol..

[199] Dranoff G. (2004). Cytokines in cancer pathogenesis and cancer therapy. Nat. Rev. Cancer.

[200] Ma Q., Long W., Xing C., Chu J., Luo M., Wang H.Y., Liu Q., Wang R.F. (2018). Cancer stem cells and immunosuppressive microenvironment in glioma. Front. Immunol..

[201] Wang S.-C., Hong J.-H., Hsueh C., Chiang C.-S. (2012). Tumor-secreted SDF-1 promotes glioma invasiveness and TAM tropism toward hypoxia in a murine astrocytoma model. Lab. Investig..

[202] Coniglio S.J., Eugenin E., Dobrenis K., Stanley E.R., West B.L., Symons M.H., Segall J.E. (2012). Microglial stimulation of glioblastoma invasion involves epidermal growth factor receptor (EGFR) and colony stimulating factor 1 receptor (CSF-1R) signaling. Mol. Med..

[203] Zanotto-Filho A., Gonçalves R.M., Klafke K., de Souza P.O., Dillenburg F.C., Carro L., Gelain D.P., Moreira J.C.F. (2017). Inflammatory landscape of human brain tumors reveals an NFκB dependent cytokine pathway associated with mesenchymal glioblastoma. Cancer Lett..

[204] Wu A., Wei J., Kong L.-Y., Wang Y., Priebe W., Qiao W., Sawaya R., Heimberger A.B. (2010). Glioma cancer stem cells induce immunosuppressive macrophages/microglia. Neuro. Oncol..

[205] Zeng F., Wang K., Huang R., Liu Y., Zhang Y., Hu H. (2019). RELB: A novel prognostic marker for glioblastoma as identified by population-based analysis. Oncol. Lett..

[206] Lee D.W., Ramakrishnan D., Valenta J., Parney I.F., Bayless K.J., Sitcheran R. (2013). The NF-κB RelB Protein Is an Oncogenic Driver of Mesenchymal Glioma. PLoS ONE.

[207] Wang H., Li L., Yin L. (2018). Silencing LncRNA LOXL1-AS1 attenuates mesenchymal characteristics of glioblastoma via NF-κB pathway. Biochem. Biophys. Res. Commun..

[208] Fedele M., Cerchia L., Pegoraro S., Sgarra R., Manfioletti G. (2019). Proneural-mesenchymal transition: Phenotypic plasticity to acquire multitherapy resistance in glioblastoma. Int. J. Mol. Sci..

[209] Boyd N.H., Walker K., Ayokanmbi A., Gordon E.R., Whetsel J., Smith C.M., Sanchez R.G., Lubin F.D., Chakraborty A., Tran A.N. (2019). Chromodomain helicase DNA-Binding Protein 7 is suppressed in the perinecrotic/ischemic microenvironment and is a novel regulator of glioblastoma angiogenesis. Stem Cells.

[210] Bhat K.P.L., Balasubramaniyan V., Vaillant B., Hummelink K., Hollingsworth F., Wani K., James J.D., Goodman L.D., Conroy S., Long L. (2013). Mesenchymal differentiation mediated by NF-κB promotes radiation resistance in glioblastoma. Cancer Cell.

[211] Singh S.K., Bhardwaj R., Wilczynska K.M., Dumur C.I., Kordula T. (2011). A complex of nuclear factor I-X3 and STAT3 regulates astrocyte and glioma migration through the secreted glycoprotein YKL-40. J. Biol. Chem..

[212] Pflug K.M. (2020). Sitcheran Targeting NF-κB-Inducing Kinase (NIK) in immunity, inflammation, and cancer. Int. J. Mol. Sci..

[213] Jung J.U., Ravi S., Lee D.W., McFadden K., Kamradt M.L., Toussaint L.G., Sitcheran R. (2016). NIK/MAP3K14 regulates mitochondrial dynamics and trafficking to promote cell invasion. Curr. Biol..

[214] Duran C.L., Lee D.W., Jung J.-U., Ravi S., Pogue C.B., Toussaint L.G., Bayless K.J., Sitcheran R. (2016). NIK regulates MT1-MMP activity and promotes glioma cell invasion independently of the canonical NF-κB pathway. Oncogenesis.

[215] Tchoghandjian A., Jennewein C., Eckhardt I., Rajalingam K., Fulda S. (2013). Identification of non-canonical NF-kB signaling as a critical mediator of smac mimetic-stimulated migration and invasion of glioblastoma cells. Cell Death Dis..

[216] Didonato J.A., Mercurio F., Karin M. (2012). NF-κB and the link between inflammation and cancer. Immunol. Rev..

[217] Verhagen A.M., Ekert P.G., Pakusch M., Silke J., Connolly L.M., Reid G.E., Moritz R.L., Simpson R.J., Vaux D.L. (2000). Identification of DIABLO, a mammalian protein that promotes apoptosis by binding to and antagonizing IAP proteins. Cell.

[218] Du C., Fang M., Li Y., Li L., Wang X. (2000). Smac, a mitochondrial protein that promotes cytochrome c-dependent caspase activation by eliminating IAP inhibition. Cell.

[219] Tchoghandjian A., Jennewein C., Eckhardt I., Momma S., Figarella-Branger D., Fulda S. (2014). Smac mimetic promotes glioblastoma cancer stem-like cell differentiation by activating NF-κB. Cell Death Differ..

[220] Eckhardt I., Roesler S., Fulda S. (2013). Identification of DR5 as a critical, NF-κB-regulated mediator of Smac-induced apoptosis. Cell Death Dis..

[221] Robe P.A., Martin D.H., Nguyen-Khac M.T., Artesi M., Deprez M., Albert A., Vanbelle S., Califice S., Bredel M., Bours V. (2009). Early termination of ISRCTN45828668, a phase 1/2 prospective, randomized study of sulfasalazine for the treatment of progressing malignant gliomas in adults. BMC Cancer.

[222] Perrot-Applanat M., Vacher S., Toullec A., Pelaez I., Velasco G., Cormier F., Saad H.E.S., Lidereau R., Baud V., Bièche I. (2011). Similar NF-κB Gene Signatures in TNF-α treated human endothelial cells and breast tumor biopsies. PLoS ONE.

[223] Szulzewsky F., Arora S., de Witte L., Ulas T., Markovic D., Schultze J.L., Holland E.C., Synowitz M., Wolf S.A., Kettenmann H. (2016). Human glioblastoma-associated microglia/monocytes express a distinct RNA profile compared to human control and murine samples. Glia.

